# Systematic review of the receptor tyrosine kinase superfamily in neuroblastoma pathophysiology

**DOI:** 10.1007/s10555-021-10001-7

**Published:** 2021-10-30

**Authors:** Esteban Javier Rozen, Jason Matthew Shohet

**Affiliations:** 1 Department of Pediatrics, UMass Chan Medical School, Lazare Research Building LRB603, 364 Plantation Street, Worcester, MA 01605 USA; 2 Division of Hematology/Oncology, Department of Pediatrics, UMass Chan Medical School, Lazare Research Building LRB603, 364 Plantation Street, Worcester, MA 01605 USA

**Keywords:** RTK, Kinase inhibitor, Neuroblastoma, RET, DDR2

## Abstract

**Background:**

Neuroblastoma is a devastating disease accounting for 15% of all childhood cancer deaths. Yet, our understanding of key molecular drivers such as receptor tyrosine kinases (RTKs) in this pathology remains poorly clarified. Here, we provide a systematic analysis of the RTK superfamily in the context of neuroblastoma pathogenesis.

**Methods:**

Statistical correlations for all RTK family members’ expression to neuroblastoma patient survival across 10 independent patient cohorts were annotated, synthesized, and ranked using the R2: Genomics Analysis and Visualization Platform. Gene expression of selected members across different cancer cell lines was further analyzed in the Cancer Cell Line Encyclopedia, part of the Cancer Dependency Map portal (depmap portal (http://depmap.org)). Finally, we provide a detailed literature review for highly ranked candidates.

**Results:**

Our analysis defined two subsets of RTKs showing robust associations with either better or worse survival, constituting potential novel players in neuroblastoma pathophysiology, diagnosis, and therapy. We review the available literature regarding the oncogenic functions of these RTKs, their roles in neuroblastoma pathophysiology, and potential utility as therapeutic targets.

**Conclusions:**

Our systematic analysis and review of the RTK superfamily in neuroblastoma pathogenesis provides a new resource to guide the research community towards focused efforts investigating signaling pathways that contribute to neuroblastoma tumor establishment, growth, and/or aggressiveness and targeting these druggable molecules in novel therapeutic strategies.

**Supplementary Information:**

The online version contains supplementary material available at 10.1007/s10555-021-10001-7.

## Introduction

### The receptor tyrosine kinase superfamily

**Fig. 1:**
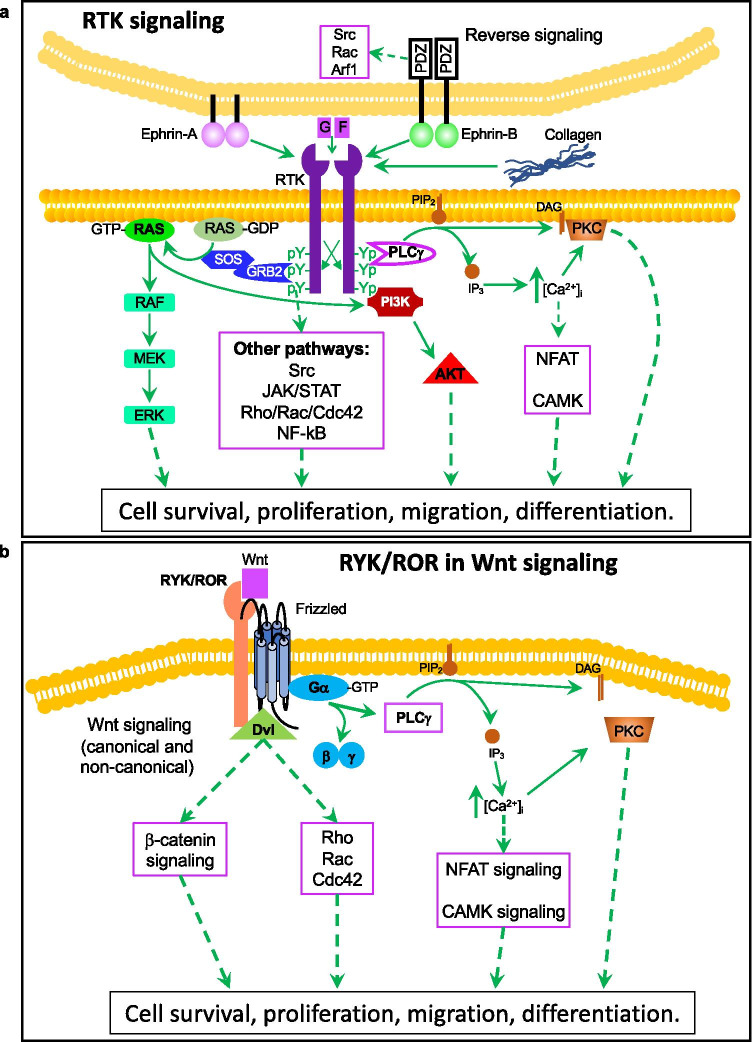
RTK-dependent signaling pathways. **a** RTK signaling. Ligands of different nature bind to their cognate RTK triggering a battery of classical and context-dependent signal transduction cascades to fulfil a wide range of cellular functions (see main text for details). **b** RYK/ROR in Wnt signaling. RYK, ROR, and a few other RTK members act as receptors for the Wnt family of ligands, leading to both canonical and non-canonical Wnt-signaling and diverse biological outputs (see main text for details). GF, growth factor (or other soluble ligands); DAG, diacylglycerol; IP_3_, inositol-1,4,5-triphosphate; PIP_2_, phosphatidyl-inositol-4,5-bisphosphate; [Ca^2+^]_i_, intracellular calcium ion concentration

The human genome comprises 58 known receptor tyrosine kinase (RTK) genes, further classified into 20 families. Members of this superfamily are involved in virtually every aspect of cellular and organismal life, including cell survival/apoptosis, cell growth and proliferation, metabolism, migration, cell cycle progression, and differentiation [1, 2]. All RTKs share a similar domain organization, composed of a specific extracellular region, harboring the ligand-binding domain, a single transmembrane helix, and an intracellular motif containing the tyrosine kinase (TK) domain. Most also contain additional juxta-membrane and C-terminal regulatory/effector motifs (Fig. 1A). RTKs are generally activated by receptor-specific ligands, such as soluble ligands/growth factors (GF), adjacent cell surface-bound ligands (ephrins), or extracellular matrix (EM) components (e.g., collagen for DDR1/2), which interact with the ligand binding domain of their cognate RTKs. This leads to dimerization or oligomerization, and activation of the kinase domain, with subsequent trans-autophosphorylation on specific tyrosine residues within the RTK’s C-terminal tail [3]. These phosphorylated tyrosine residues in turn act as docking sites for the recruitment and activation of adaptor and effector proteins to propagate and amplify the signals through different transduction cascades. These include the classical PLCγ/Ca^2+^/PKC, Ras/MAPK, and PI3K/AKT pathways or the more context-specific JAK/STAT, Rho family of GTPases, or Src family of kinases, among others [4, 5] (Fig. 1a). A deviation from this general mechanism of signaling is documented for a subset of RTKs, namely RYK, ROR1/2, PTK7, and MUSK. Except for the later, these RTKs are classified as pseudokinases, as they lack a kinase activity. Instead, this group of RTKs act as co-receptors for WNT family ligands, triggering both canonical and non-canonical WNT-mediated signaling pathways (Fig. 1b; reviewed in [6]). The overall topology of RTKs, their mechanisms of activation, and downstream intracellular signaling components are highly conserved throughout the evolution from nematodes to humans. Therefore, it is not surprising that dysregulation of RTK signaling leads to many human diseases including cancer (reviewed in [4]).

Numerous genomic and cellular studies have revealed many types of context-specific pathogenic alterations in RTK genes including oncogenic mutations and epigenetic modifications of *EGFR*, *HER2*, and *MET*, among many others [4]. Such studies have led to the development of novel therapeutic compounds that block or attenuate RTK signaling. Noteworthy, the therapeutic efficacy of RTK inhibitors in oncology is highly dependent on the cell context and the relative contribution of different RTK signaling pathways in the development of a given cancer. In this regard, the critical roles that specific RTKs play in the initiation, metastasis, drug resistance, and relapse in neuroblastoma are just starting to emerge, although very little is yet known for the most part, warranting further efforts to increase our current knowledge on this broad topic.

### Neuroblastoma

Neuroblastoma is the most common extracranial malignant tumor in children, accounting for 7% of all pediatric neoplasms in patients under 15 years and about 13% of all pediatric cancer deaths. The biological heterogeneity of neuroblastoma results in a variety of clinical presentations. Patients with low- and intermediate-risk neuroblastoma have favorable prognosis and an excellent 5-year survival rate with surgical resection and modest chemotherapy. However, in the case of high-risk metastatic neuroblastoma survival is between 50 and 60%, despite highly genotoxic chemotherapy, radiation, and surgery (reviewed in [7]). Efforts to improve survival and reduce long-term impact of treatment will require novel therapeutics such as innovative application of small molecule RTK inhibitors, epigenetic targeting, and other neuroblastoma context-specific interventions.

While neuroblastoma tumorigenesis is thought to arise from the disrupted development of sympatho-adrenergic precursors of the neural crest, no common genetic or epigenetic alteration has been found to account for most cases of neuroblastoma [8]. Structural genomic changes found in subsets of neuroblastoma, and linked to tumorigenesis and reduced survival, include *MYCN* amplification, *ALK* activating mutations, 1p36 deletion, or 17q gain [9, 10]. The *MYCN* oncogene plays a major role in neural crest development and neuroblastoma tumorigenesis and defines an aggressive subset of tumors. Amplification of *MYCN* (defined as > 10 copies) is found in about half of all high-risk tumors and confers a particularly poor prognosis. The “anaplastic lymphoma kinase” (*ALK*) gene belongs to the RTK superfamily. Activating mutations for ALK are found in 6–10% of spontaneous cases and in almost 50% cases of familial neuroblastoma (1–2% of total neuroblastoma cases) [11]. This RTK has also been implicated as an oncogene in lymphomas and lung cancers, where it is typically found as a translocated fusion gene (e.g., *ALK-NPM*) [12, 13]. Recent studies link *ALK* to sympathetic neuron development and survival of migratory neural crest cells [14]. This gene is a direct transcriptional target of MYCN and is an important regulator of stemness, including STAT3-dependent self-renewal [15, 16]. Recent data from genetically engineered mouse models of neuroblastoma confirm that ALK and MYCN cooperate to promote tumorigenesis [17]. Importantly, potent ALK inhibitors are already in clinical trials for ALK-mutant neuroblastomas.

While some of the roles that ALK and a few other RTK family members play in neuroblastoma initiation, progression, or aggressiveness have been examined, the rationale for such studies has usually been linked to known functions of such genes in other cancer paradigms. This may not necessarily reflect a specific tumorigenic function in the context of neuroblastoma pathogenesis. Despite the functions of several RTKs have been widely studied in the context of neural crest differentiation (reviewed in [18]), a systematic unbiased review on the contribution of the RTK superfamily to neuroblastoma pathogenesis is lacking.

In order to establish a rationale for the systematic analysis of the RTK superfamily in neuroblastoma, we initially annotated, synthesized, and ranked the correlation between overall survival probability and gene expression for all 58 members of the RTK superfamily (sorted by phylogenetic order) across ten independently annotated patient cohorts (Cangelosi, Kocak, SEQC-RPM, SEQC-custom, Primary NRC, Oberthuer, TARGET-Asgharzadeh, Seeger, Maris, and Versteeg) with available patient survival data in the R2: Genomics Analysis and Visualization Platform (http://r2.amc.nl) [19] (Supplementary Fig. 1; see Fig. 3a for a representative example of the correlation plots from which the adjusted p values were taken).

**Fig. 2:**
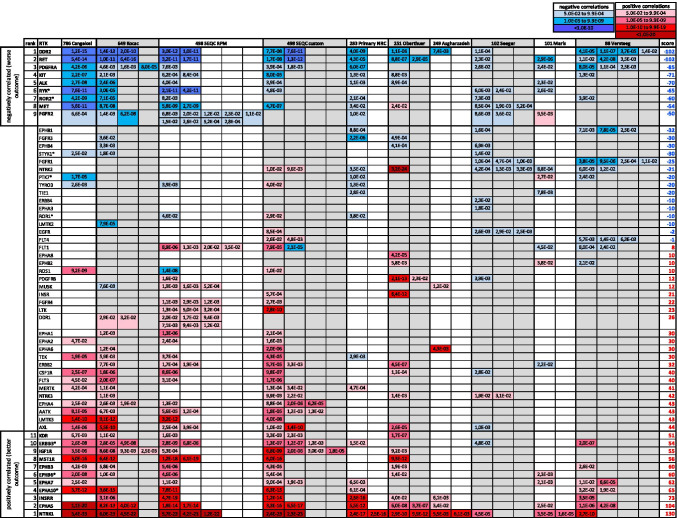
Systematic analysis of RTK expression correlation to neuroblastoma patient survival. Association of RTK gene expression to neuroblastoma patient survival sorted by score. Significant Bonferroni-adjusted *p* values (*p* value < 0.05) from the correlation of neuroblastoma patient survival probability to RTK gene expression for every member of the RTK superfamily (58) across 10 publicly available datasets were annotated and ranked (see main text and Methods section for details). The figure is presented as a pseudo-heatmap table, highlighting the most significant negatively (top subset; blue) and positively (bottom subset; red/pink) correlated RTK members. Top subset: RTKs with − 50 points or less, corresponding to RTKs that show a significant adjusted *p* value in 5 or more independent datasets correlating to reduced patient survival. Bottom subset: RTKs with 50 points or more, corresponding to RTKs that show a significant adjusted *p* value in 5 or more independent datasets correlating to increased patient survival. * Asterisks denote members known or predicted to be pseudokinases

Re-sorting of the RTK superfamily gene list by score (Fig. 2) clearly defined two subsets of RTKs showing significant associations to either worse (Fig. 2 top subset) or better (Fig. 2 bottom subset) prognosis across at least 5 independent cohorts, thus implying a potential role for each of these RTKs as critical regulators of neuroblastoma pathogenesis. Among the RTKs showing a robust positive correlation (higher expression = higher survival probability), we found—in order of ranking—NTRK1, EPHA5, INSRR, EPHA10*, EPHA7, EPHB3, EPHB6*, MST1R, IGF1R, ERBB3*, and KDR (Fig. 2 bottom subset). Asterisks denote members that are classified as pseudokinases, since they exhibit minimal or no kinase activity. Despite lacking enzymatic function, pseudokinases display regulatory and/or signaling roles via alternative mechanisms (reviewed in [20]). Importantly, the roles for some of these RTKs in promoting neuronal differentiation in general and better neuroblastoma prognosis (e.g., NTRK1, INSRR, EPHB6, and ERBB3) have been previously established, thus confirming the reliability of our approach. Importantly, we also identified multiple RTKs with no previous link to neuroblastoma tumorigenesis or known association with clinical outcome, thus constituting novel and potentially valuable markers for classification and/or prognostic strategies.

### RTKs associated to a better outcome

Receptor tyrosine kinases showing consistent correlations to better patient survival probability may be useful prognostic markers in neuroblastoma and indicate pathways involved in neural crest differentiation. This is the case of NTRK1/TrkA, whose expression and functions have extensively been associated to neuroblastoma spontaneous regression/differentiation and to better outcomes (reviewed in [21, 22]). Expressions of EPHB6 [23, 24] and ERBB3 [25, 26] have also been previously associated with better prognosis. On the other hand, a number of studies have characterized the pro-oncogenic activities of IGF1R in neuroblastoma cell lines and tumor models [27–30]. Therefore, it was very surprising to find a very robust correlation of IGF1R expression to a better survival probability in 5 independent patient datasets. Among the several cellular functions induced by this RTK in neuroblastoma cells, a major one is cell differentiation [31], suggesting that under specific *in vivo* contexts, IGF1R suppressive activities might dominate over pro-oncogenic activities observed in cell culture. The related INSRR RTK also exhibited a strong positive correlation to survival in 7 out of 10 cohorts, ranking in the 3rd position overall. The *INSRR* genetic locus is found in the opposite DNA strand of *NTRK1*. Interestingly, these genes have promoters and transcription start sites less than 2 kb apart in a head-to-head orientation [32]. Furthermore, expression of both genes has been shown to be co-regulated in neural crest-derived neurons [33] and in neuroblastoma tumor samples [34].

Finally, KDR (or VEGFR2) is an established proto-oncogene in several solid tumor models, including neuroblastoma [35, 36], due to its central role orchestrating tumor neo-angiogenesis. Thus, our finding of its genetic association with improved outcome is unexpected. One possible explanation for this might rely on the alternative splicing variant sVEGFR2, which acts as a secreted endogenous inhibitor of angiogenesis. In this context, Becker et al. [37] demonstrated MYCN-dependent downregulation of sVEGFR2 in advanced stages neuroblastoma tumor samples. Thus, in low-risk neuroblastoma patients, higher expression of VEGFR2 (in the form of the sVEGFR2 variant) might indeed be correlated to a better survival probability. Alternatively, expression of the ligand VEGF has also been correlated to neuroblastoma differentiation and to a favorable prognosis in neuroblastoma patients [38], suggesting a neuroblastoma cell-intrinsic tumor-suppressive role for VEGF/KDR signaling, independently of its angiogenic function on endothelial cells. Overall, the roles of KDR-mediated signaling in neuroblastoma cell homeostasis await future elucidation.

To the best of our knowledge, there are no current reports associating *EPHA5*, *EPHA10*, *EPHA7*, *EPHB3*, or *MST1R* to neuroblastoma pathogenesis. Interestingly, the fact that 5 different members of the EPH RTK subfamily were represented within the top 7 scores of our analysis points to a likely redundant but essential function of this RTK family as modulators of neuroblastoma cell homeostasis and/or differentiation. Future efforts should consider the incorporation of such markers into neuroblastoma tumor diagnosis and classification protocols.

### RTKs associated to a worse outcome

Regarding RTKs that showed a significant negative correlation (higher expression = lower survival probability) across at least 5 cohorts, we found RET, DDR2, PDGFRA, KIT, ALK, RYK*, ROR2*, MET, and FGFR2 (Fig. 2 top subset). Once more, the fact that some of such RTKs have previously been described as important players in neuroblastoma tumor biology (e.g., ALK, KIT, MET, and RET) again validates this approach. More importantly, RTKs that have remained poorly studied in the context of neuroblastoma pathogenesis, such as DDR2 and PDGFRA, are now revealed as very robust and potentially critical modulators of neuroblastoma.

Our systematic analysis is based on correlations of patient survival probability to mRNA expression of the different RTK superfamily members. It is well known that in many cases, mRNA levels do not necessarily reflect protein abundance [39, 40]. In recent years, several proteomic and phosphoproteomic studies on neuroblastoma cells and models have been carried out, contributing to our better understanding of the kinases and pathways underlying this malignancy. On this regard, DeNardo et al. [41] performed a quantitative phosphoproteomic profiling of the neuroblastoma cell line NB10 versus a neural progenitor cell line (NPC) as a control. This analysis demonstrated significant enrichment of phosphorylated RTK peptides and downstream mediators of signaling in the NB10 cells. Among such phosphorylated RTKs, they found—in order of relative increase over the control—RET, DDR2, ROR2, IGF1R, EPHA2, and FGFR2. Subsequent work including phosphoproteomic and sophisticated informatic analyses of protein–protein interaction on 4 neuroblastoma cell lines under different experimental conditions [42] identified significant enrichment of phosphorylated peptides from several RTKs in the endosomal compartment on at least 2 independent cell lines. These RTKs included—in relative order of magnitude—DDR2, ALK, KIT, RET, EGFR, PDGFRA, FGFR1, IGF1R, EPHB3, EPHA2, and EPHB2. Taken together, these studies not only confirm the expression at the protein level for many of our top candidate RTKs in neuroblastoma cells, but also their relatively increased phosphorylation, which is generally associated to activation, a feature further supported by the concurrent phosphorylation of several downstream signaling components. Thus, mRNA expression does seem to reflect protein expression and function in this particular context. Future efforts should examine to which extent these observations are maintained in patient-derived tumor samples and whether there are expression and function correlations to disease stage and/or to specific cell populations within these tumors.

Given that these RTKs may constitute potential pharmacological targets for neuroblastoma treatment, an in-depth review of the current literature on such receptors will be the focus of the following sections. Our new identification of RTKs whose expression strongly correlates with poor neuroblastoma survival across multiple independent clinical cohorts (Fig. 2 top subset) opens new avenues for development of novel therapeutic interventions targeting these receptors. Below we review the current literature and detail potential context-dependent oncogenic functions of the top ranked RTKs from our analysis.

#### RET

**Fig. 3:**
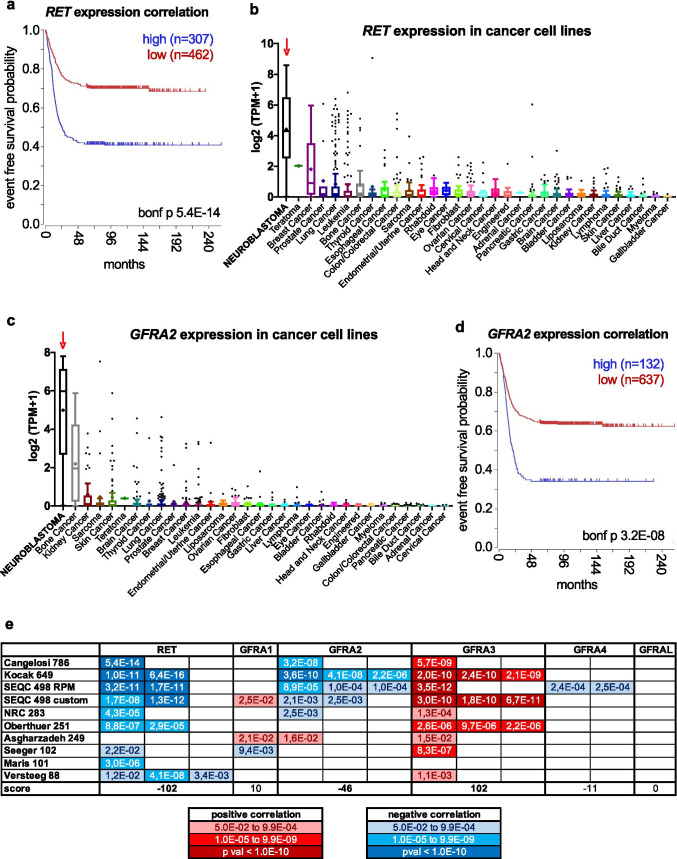
RET signaling components in neuroblastoma patient samples and cell lines. **a** Representative Kaplan–Meier plot for the correlation of *RET* expression to patient survival probability from R2 (Cangelosi 786 cohort). *RET* was the top negatively correlated RTK (Fig. 2 top subset), indicative of its role in NB pathogenesis and aggressiveness (as previously shown by others; see main text for details). “bonf p” = Bonferroni adjusted *p* value. **b** *RET* mRNA expression across different cancer cell lines (CCLE) showing a high and specific average *RET* expression in neuroblastoma cell lines (red arrow) as compared to all other cancer cell lines. **c** *GFRA2* mRNA expression across different cancer cell lines (CCLE) showing a high and specific average expression in neuroblastoma cell lines (red arrow) as compared to all other cancer cell lines. **d** Representative Kaplan–Meier plot for the correlation of *GFRA2* expression to patient survival probability obtained from R2 (Cangelosi 786 cohort). “bonf p” = Bonferroni adjusted p value. **e** Association of RET co-receptors mRNA expression to neuroblastoma patient survival. Significant Bonferroni-adjusted *p* values (*p* value < 0.05) correlating patient survival probability to *GFRA* co-receptors across 10 publicly available datasets were annotated and ranked (see Methods and Fig. 2 for details). The panel is presented as a pseudo-heatmap table

The proto-oncogene *RET* (“rearranged during transfection”) encodes an RTK for the glial cell line-derived neurotrophic factor family of ligands (GDNF, NRTN, ARTN, PSPN, and GD15). Ligand-receptor binding and specificity is afforded by one of five “GDNF family receptor alpha” (GFRα1, GFRα2, GFRα3, GFRα4, and GFRαL) co-receptors. The GDNF/RET signaling axis plays a major role during sympathetic and enteric nervous systems development, where it mediates proliferation, migration, and differentiation (reviewed in [43–45]). RET is an oncogene and constitutive activation results in human multiple endocrine neoplasia (MEN) syndromes 2A and 2B and familial medullary thyroid carcinoma [46]. Pioneer studies reporting RET expression and function in neuroblastoma models date back to more than 30 years ago [47–49], but the actual implications of this kinase in neuroblastoma pathogenesis have recently started emerge. Initially, Iwamoto et al. [50] reported neuroblastoma development in a transgenic mouse that carried the *RET* oncogene driven by a mouse metallothionein regulatory element. Additional reports using cell lines support a role for GDNF/RET signaling in differentiation, migration, and metastasis [51–56]. Recently, using neuroblastoma models carrying activating *Alk* mutations in the context of MYCN overexpression, Cazes et al. [57] demonstrated that RET is upregulated in an ALK-dependent fashion. RET inhibition by the small-molecule vandetanib (ZD-6474) significantly reduced tumor growth in this genetically engineered mouse model (GEMM). In a follow-up study from the same group [58], an oncogenic *RET* mutation in the context of the TH-MYCN mouse model recapitulated the phenotype seen in mutant *ALK*/TH-MYCN mice. Furthermore, a synergistic *in vivo* antitumor effect was shown by concomitant RET (vandetanib) and ALK inhibition (crizotinib). In parallel, Zhang et al. [59] characterized the effect of another RET inhibitor, cabozantinib (XL184), on *in vitro* and *in vivo* neuroblastoma paradigms. Subsequent studies from the same group found comparable results when using the small-molecule RET/multikinase inhibitor RXDX-105 [60] or the RET/multikinase inhibitor regorafenib (BAY 73–4506) [61]. Notably, the effect of regorafenib on *in vitro* and *in vivo* neuroblastoma models had been previously reported by Chen et al. [62]. Moreover, regorafenib was clearly established as a RET signaling inhibitor in neuroblastoma cell lines, and its tumor suppressant effect was validated *in vivo* in a xenograft tumor system and in the immuno-competent TH-MYCN transgenic mouse model. In our analysis, RET (together with DDR2) ranked as the top RTK whose expression robustly correlated to an unfavorable outcome across the majority of neuroblastoma patient cohorts (Fig. 2 top subset and Fig. 3a). We additionally analyzed RET expression across 1378 cancer cell lines from the Cancer Cell Line Encyclopedia (depmap portal) [63–65] and found that neuroblastoma cell lines display the highest mean *RET* mRNA expression (Fig. 3b).

Despite the findings above, other groups have presented data pointing to a role for RET in neuroblastoma differentiation in the context of retinoic acid (RA)-induced differentiation [66–72]. In this line, RET is proposed to act as a tumor suppressor by promoting a transcriptional program leading to a terminally differentiated (non-malignant) neuronal phenotype. It is worth noting that most of these analyses have been performed in the context of *in vitro* cell differentiation assays with specific neuroblastoma cell lines. While more physiologically relevant models await investigation, a possible explanation might rely on the cell context-dependent expression of GFRα co-receptors. *GFRA2* is highly and specifically expressed in neuroblastoma cell lines (Fig. 3c and [73]) and promotes their proliferation [73]. Additionally, GFRα2 has been proposed as a potential target for antibody-/CAR T cell-based therapies against neuroblastoma [74]. Lastly, *GFRA2* (Fig. 3d–e) and its ligand *NRTN* (not shown) both showed significant association to reduced survival in 5 independent patient datasets, while *GFRA3* exhibited the opposed correlation in 9 out of 10 cohorts (Fig. 3e). Therefore, a cell-type specific GFRα2/GFRα3 ratio could indeed determine the tumor promoting vs. tumor suppressing behavior of RET. This hypothesis is further supported by expression correlation data across 12 publicly available neuroblastoma datasets [75], which showed significantly positive associations of *RET* with *GFRA2*, but negative in the case of *GFRA3*. Other signaling components could also contribute to this decision. For instance, the scaffold protein PAG1 can modulate the localization and function of the Src family kinases FYN and LYN and ultimately help finetune the proliferation vs. differentiation behavior of neuroblastoma cells [76, 77]. Altogether, these conflicting results highlight the need for careful tumor modeling and appreciation of the context-dependent roles of RTKs in general.

In summary, a growing body of literature suggests that RET plays an important role promoting neuroblastoma tumorigenesis and aggressive phenotypes. As described above, several groups have started to focus on targeting RET signaling as a novel strategy against neuroblastoma tumors, showing promising results that demand further efforts and clarification.

#### DDR2

The discoidin domain receptor family of RTKs is composed by 2 members, DDR1 and DDR2. The natural ligand for DDR receptors is collagen, and upon ligand binding, these RTKs become activated with slow and sustained kinetics [78, 79]. DDR receptors are implicated in many developmental and physiological roles, such as mammary gland development (for DDR1 [80]) or proper bone growth (for DDR2 [81]), while their deregulation may underlay certain types of cancer (reviewed in [82–85]). Somatic mutations of DDR2 are present in 3–4% of patients with squamous cell lung carcinoma [86] and at comparable frequencies in cervical carcinoma, melanoma, colorectal cancer, and some head and neck cancers [82, 87, 88]. The best understood oncogenic role of DDRs is their involvement in tumor invasion and metastasis. DDRs participate in several steps of the metastatic process including activation of epithelial to mesenchymal transition (EMT), and by promoting cell migration via degradation of the extracellular matrix and tissue colonization [83].

**Fig. 4:**
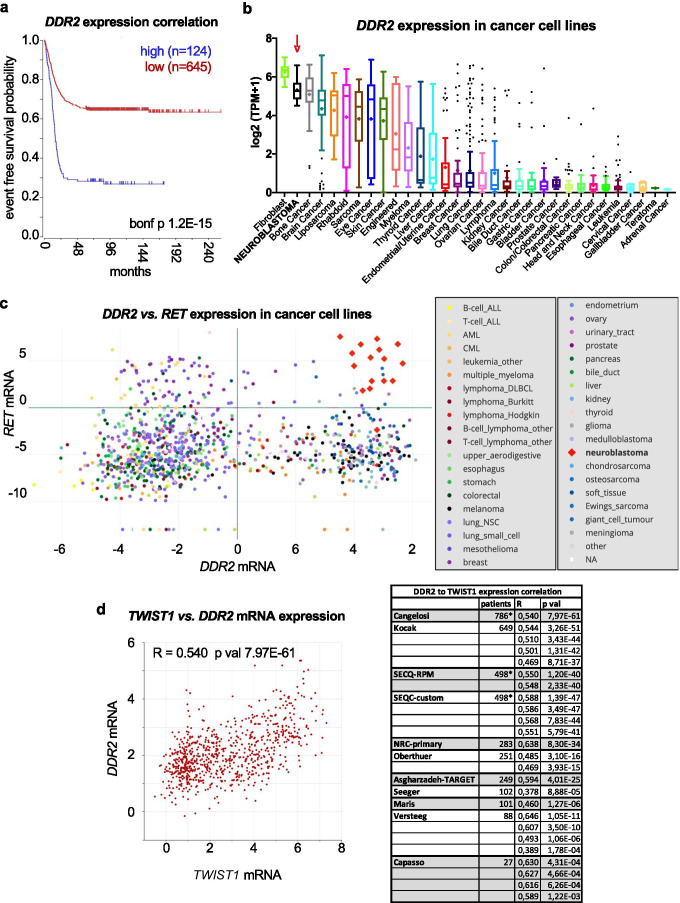
*DDR2* expression in neuroblastoma patient tumor samples and cell lines. **a** Representative Kaplan–Meier plot for the correlation of *DDR2* expression to patient survival probability obtained from R2 (Cangelosi 786 cohort). “bonf p” = Bonferroni adjusted *p* value. **b** *DDR2* mRNA expression across different cancer cell lines (CCLE) showing a relatively high average expression (top 2) in neuroblastoma cell lines (red arrow) as compared to all other cancer cell lines. **c** Expression of *RET* vs. *DDR2* mRNA (log2(TPM + 1)) across cancer cell lines (CCLE), demonstrating a high and exclusive co-expression of both markers in neuroblastoma cell lines. **d** *DDR2* and *TWIST1* expression are highly correlated in every neuroblastoma patient dataset. *Left panel*: representative plot of *DDR2* and *TWIST1* expression correlation analysis from R2 (Cangelosi 786 cohort). *Right panel*: correlation coefficients (R) and corresponding *p* values for all available *DDR2* to *TWIST1* expression associations from 11 independent patient datasets (R2), predicting a transcriptional/functional co-dependency, as previously described in other models (see main text for details). * Asterisks denote datasets containing the same patient cohort but analyzed by different techniques or analytical methods

Our present analysis establishes a highly significant and robust correlation between *DDR2* expression and neuroblastoma patient outcome in 9 out of 10 patient datasets, ranking on the top of our list together with RET (Fig. 2 top subset and Fig. 4a). We further investigated *DDR2* expression in the Cancer Cell Line Encyclopedia (CCLE) and observed that neuroblastoma cell lines ranked in the top 2nd position for highest average expression (Fig. 4b). Moreover, high co-expression of *RET* and *DDR2* distinguishes neuroblastoma cell lines from virtually any other cancer cell line in the CCLE (Fig. 4c). Despite of this, we did not observe a very high expression correlation between DDR2 and RET in the R2 tumor datasets (not shown), which could suggest expression in different tumor cell subpopulations. This idea is further supported by recent transcriptional characterization of paired mesenchymal vs. adrenergic isogenic cells from human neuroblastoma tumors [89]. This work established a mesenchymal gene signature set of 485 mRNAs and a 369-adrenergic gene signature. Importantly, *DDR2* was one of the mesenchymal signature genes, while *RET* and *ALK* were listed in the adrenergic set. In line with this, Siaw et al*.* recently showed that loss of *RET* promotes mesenchymal identity in neuroblastoma cells [56]. Moreover, the authors also observed that RET expression and activity are directly regulated by ALK.

Interestingly, *DDR2* is a key transcriptional target of TWIST1 during cranial mesoderm development [90] and in ovarian cancer cells, where TWIST1-dependent *DDR2* expression was critical for EMT, migration, invasion, and *in vivo* metastasis [91]. The TWIST1 transcription factor is a master regulator of EMT and metastasis (reviewed in [92]). Moreover, DDR2 activation promotes SNAIL1 protein stabilization and EMT of metastatic breast cancer cells [93]. It is worth noting that in neuroblastoma, *TWIST1* is a direct transcriptional target of MYC/MYCN [94]. In further analyses on the R2 Genomics Platform, we found very significant correlations between *DDR2* and *TWIST1* expression in every single neuroblastoma patient dataset available (Fig. 4d). Hence, it can be hypothesized that the transcriptional axis MYCN/TWIST1/DDR2/SNAIL1 could drive high-risk neuroblastoma tumor invasion and metastasis. Finally, our analysis and literature review suggest that DDR2 may be an excellent therapeutic target in neuroblastoma, a feature that is currently being addressed in our lab.

#### PDGFRA

The platelet-derived growth factor receptor A (or CD140a) is an RTK expressed in a wide spectrum of cell types, exerting a range of developmental and physiological functions in mesodermal-derived tissues, such as connective tissue, blood, and mesangial cells [95, 96]. Importantly, PDGFRA signaling has been shown to contribute to the development of cranial and cardiac neural crest-derived structures (reviewed in [18]). The roles of this RTK in cancer have been extensively studied in the context of gastrointestinal stromal tumors (GIST) where activating point mutations on *PDGFRA* are found in 5–7% of GIST cases [97] (reviewed in[98]). *PDGFRA* rearrangements (mostly *FIP1L1-PDGFRA* fusions) are found in 10–20% of patients with idiopathic hypereosinophilia and in some cases of systemic mastocytosis [99]. Finally, amplifications of the *PDGFRA* gene are observed in 5–10% cases of glioblastoma multiforme. *PDGFRA* amplification has also been observed in oligodendrogliomas [100], esophageal squamous cell carcinoma [101], and arterial intimal sarcomas [102]. Thus, overexpression and/or hyperactivation of PDGFRA plays a role in the pathogenesis of multiple tumor types (reviewed in [98]).

In relation to neuroblastoma, Matsui et al. [103] reported the expression of *PDGFRA* transcripts and protein in several neuroblastoma cell lines, although Beppu et al. [104] was only able to detect PDGFRA expression in 1 (SH-SY5Y) out of 7 neuroblastoma lines. Also, a potential role for PDGFRA has been suggested during *in vitro* differentiation of SH-SY5Y cells towards a neuronal phenotype [105]. In our analysis of expression correlation, *PDGFRA* ranked in the 3rd top position, showing statistically significant correlation with poor survival in 8 out of 10 cohorts (Fig. 2 top subset and Supplementary Fig. 2a). Furthermore, analysis of *PDGFRA* mRNA expression in the Cancer Cell Line Encyclopedia showed that neuroblastoma cell lines express relatively high levels of this RTK, ranking in 2nd position in average overall (Supplementary Fig. 2b). Therefore, and given the established roles for this receptor in other tumor types, our data implicates this RTK as a potential therapeutic target in neuroblastoma. Several relatively non-specific inhibitors of PDGFRA (i.e., imatinib and sunitinib) are currently in clinical trials, and as more specific and potent PDGFRA inhibitors are developed, our findings support preclinical testing in neuroblastoma tumor models.

#### KIT

The *KIT* proto-oncogene (c-KIT aka CD117) encodes the RTK for the stem cell factor (SCF) ligand. SCF/c-KIT signaling contributes to organ development and homeostasis, in part by maintaining the stemness of progenitor cells in several adult tissues [106, 107]. Although KIT activating mutations have been found to drive transformation in GIST, acute myeloid leukemia, mast cell leukemia, and melanoma, no gain-of-function mutations on KIT have been reported in neuroblastoma [108] [109]. Initial studies from Cohen et al. [110] analyzed SCF/KIT expression in neuroblastoma tumor samples and cell lines. This and subsequent reports [111–113] confirmed a role for SCF/KIT signaling driving cell growth and proliferation. Vitali et al. [114] established a positive correlation between KIT and MYCN expression and found that KIT-mediated proliferation *in vitro* was sensitive to the multikinase inhibitor imatinib. Moreover, Uccini et al. [115] observed that in primary neuroblastoma tumor samples, SCF/KIT expression correlated to MYCN amplification, and worse overall survival. A small subpopulation of cancer stem cells characterized by high expression of c-KIT was detected from several tumor samples and cell lines [116]. This was further investigated by Lau et al. [117], who demonstrated that KIT^+^ cells are generated de novo during neuroblastoma progression and may contribute to neuroblastoma proliferation. KIT^+^ cells expressed higher levels of neural crest and stem cell markers and displayed a more aggressive phenotype and *in vivo* self-renewal capacity compared to KIT^−^ subpopulations. However, other reports suggest that expression of KIT statistically correlated to a better prognosis in neuroblastoma tumor samples [118, 119]. Overall, our expression-to-survival correlation data (Fig. 2 top subset), together with several recent studies, suggest a role for this RTK as a relevant mediator of neuroblastoma tumor progression and resistance/relapse. Further studies on KIT functions in neuroblastoma and the impact of specific inhibitors on tumor stemness and metastasis are warranted.

#### ALK

The “anaplastic lymphoma tyrosine kinase” receptor, ALK, is altered by gain-of-function point mutations in half of familial neuroblastoma cases (~ 1% of total cases), and in around 9% of sporadic neuroblastoma, climbing up to 14% in high-risk patients [11, 120–122]. As mentioned above, the roles of ALK in neuroblastoma pathogenesis have been extensively characterized, with several reviews published in recent years. Hence, for further reading on this specific topic, the reader is kindly referred to [123–125].

#### RYK and ROR2

The RYK, ROR, PTK7, and MUSK families share a unique characteristic by serving as receptors for ligands of the WNT family (Fig. 1b) [126–129]. Except for MUSK, all of them are classified as pseudokinases. The function of these WNT-binding receptors is essential for several developmental processes (for complete reviews on RYK/ROR2 biology in health and cancer, see [6, 130, 131]). Increasing studies implicate these pseudokinases in many aspects of tumor physiology including self-renewal, migration/metastasis, and drug resistance [6]. Although most of such data derive from *in vitro* models using cancer cell lines, *in vivo* experimental data supporting oncogenic roles for ROR2 have been reported in melanoma [132], renal cell carcinoma [133], ovarian cancer [134], and breast cancer [135], as well as for RYK in gastric [136] and ovarian cancer [137].

**Fig. 5:**
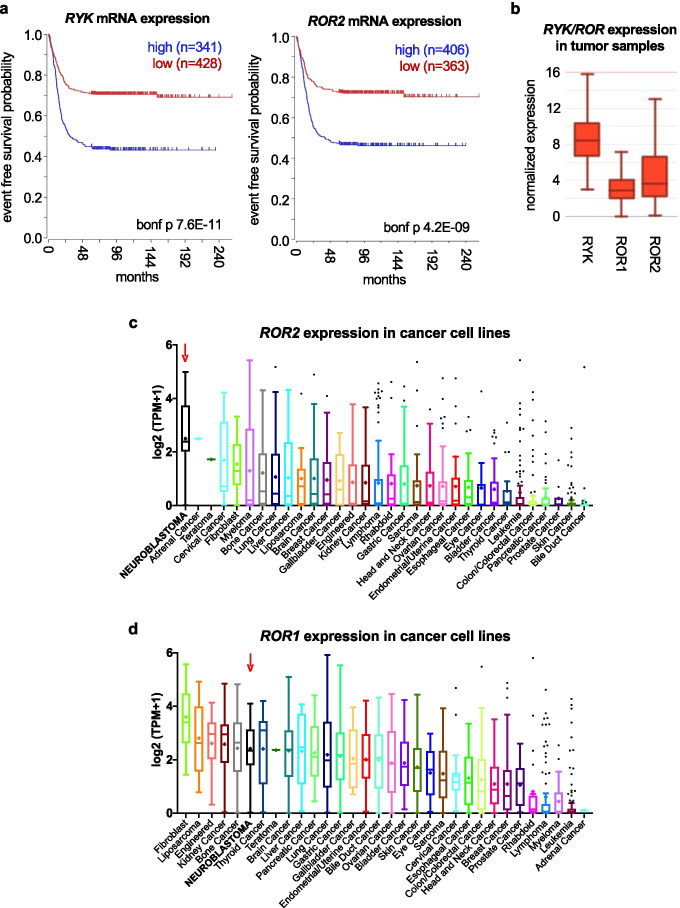
*RYK* and *ROR2* expression in neuroblastoma patient samples and cell lines. **a** Representative Kaplan–Meier plots for the correlation of *RYK* and *ROR2* expression to patient survival probability obtained from R2 (Cangelosi 786 cohort). “bonf p” = Bonferroni adjusted *p* value. **b** Representative boxplot showing average expression of *RYK*, *ROR1*, and *ROR2* in a neuroblastoma patient dataset (Cangelosi 786) from the R2 platform. Note the relatively higher expression of *RYK* (similarly observed in several cohorts; Supplementary Fig. 2C). **c** Expression across different cancer cell lines from the CCLE showing a relatively high and specific expression of *ROR2* in neuroblastoma cell lines (top average expression overall). **d** Expression of *ROR1* mRNA across different cancer cell lines from the CCLE displaying less specific expression

Surprisingly, while the roles for WNT signaling in neuroblastoma have been extensively studied (for recent reviews, see [138, 139]), how RYK and ROR2 modulate WNT in neuroblastoma remains largely unexplored. RYK can act as a receptor for WNT5A and other WNT family ligands that drive neural crest migration and differentiation. One study demonstrated that *RYK* mRNA was detected across a panel of 25 neuroblastoma cell lines [140]. The RTK genes *ROR1* and *ROR2* were originally cloned form the neuroblastoma cell line SH-SY5Y [141]. Wnt/Ror2 signaling modulates the migration of neural crest cells during Xenopus development [142] and is hypothesized to a play similar roles in mammals [140]. Recently, Dave et al. confirmed ROR1 and ROR2 expression in neuroblastoma cell lines and patient-derived tumor samples [143]. This group showed that anti-ROR1 antibodies could target neuroblastoma cells for natural killer (NK)-mediated cell killing, while CAR T cells targeting ROR1^+^ neuroblastoma cells are currently under development [144]. Our analysis of gene expression from neuroblastoma tumor datasets uncovered a significant association for *RYK* and *ROR2*—but not for *ROR1*—to a worse survival probability in 6 out of 10 cohorts (Fig. 2 top subset and Fig. 5a). Comparison of the relative expression for *RYK* and *ROR1/2* across clinical datasets shows overall higher average expression of *RYK*, with *ROR1* and *ROR2* showing similarly lower levels (Fig. 5b and Supplementary Fig. 2C). Furthermore, *ROR2* expression analysis in cancer cell lines (CCLE, Broad Institute) suggests a relatively high mRNA level in neuroblastoma cells, showing the highest average expression among all cancer cell lines (Fig. 5c), while *ROR1* showing low specificity for neuroblastoma cell lines (Fig. 5d). Altogether, we found that *RYK* and *ROR2*, but not *ROR1*, are robustly associated with worse outcome in neuroblastoma. This is further supported by a recent study describing a role for ROR1, but not ROR2, in retinoic acid-induced neuroblastoma differentiation [145]. Together, these data suggest that the WNT modifying pseudokinases ROR2 and RYK represent potential novel therapeutic targets for neuroblastoma treatment and that current research on immuno-therapeutic strategies against ROR1 might need to be re-evaluated in favor of ROR2 and RYK.

#### MET

MET (c-Met or HGFR) is the tyrosine kinase receptor for hepatocyte growth factor (HGF). This receptor-ligand interaction triggers classical RTK signaling pathways to promote cell migration, angiogenesis, and proliferation (reviewed in [146]). MET gain of function via overexpression, amplification, aberrant splicing, or mutations is associated with multiple cancer types, such as non-small cell lung carcinoma [147], gastrointestinal cancer [148], and hepatocellular carcinoma [149]. Regarding neuroblastoma, initial studies demonstrated a role for HGF/MET signaling in the migration and/or differentiation of neural crest cell-derived structures [150]. More direct evidence by Hecht et al. [151] characterized the expression and signaling mechanisms of HGF/MET in cultured neuroblastoma cell lines. Although they did not observe any effects on *in vitro* proliferation, the authors reported a very clear role for HGF/MET signaling in migration/invasion of neuroblastoma cells, both *in vitro* and *in vivo*. The same group also showed that NTRK2 activation preceded and mediated HGF/MET upregulation in neuroblastoma cell lines [152]. Furthermore, they also found that sublethal irradiation led to upregulation of HGF and MET in neuroblastoma cell lines, resulting in enhanced migratory behavior [153]. Analyses for MET amplification, alternatively spliced isoforms, or protein over-expression in a small cohort of neuroblastoma tumor samples (54 samples) suggested a low prevalence of such alterations [154]. However, the development of novel small molecule inhibitors targeting c-MET activity has prompted to a re-evaluation of HGF/MET signaling in neuroblastoma and other cancer paradigms. The highly selective MET inhibitor PHA665752 was shown to significantly reduce *in vitro* migration and proliferation on two neuroblastoma cell lines in a dose-dependent manner [155]. The MET inhibitor EMD1214063 was also shown to efficiently reduce *in vitro* viability and inhibit *in vivo* neuroblastoma tumor growth in orthotopic xenograft mouse models [156]. Finally, MET upregulation in tumors upon pan-VEGF inhibitor therapy may act as an escape mechanism to acquire resistance and regain neoangiogenic capabilities [157]. The MET/multikinase inhibitor cabozantinib blocked neuroblastoma cell proliferation and migration and efficiently reduced *in vivo* neuroblastoma tumor growth and metastases in orthotopic xenograft mouse models [158]. We found a strong correlation between *MET* expression and a lower neuroblastoma patient survival probability in 6 out of 10 datasets (Fig. 2 top subset). In summary, our analysis together with published data highlight the need of future efforts to advance our understanding on the regulation and functions of this RTK in several aspects of neuroblastoma pathophysiology.

#### FGFR2

The *FGFR2* gene (fibroblast growth factor receptor 2) can give rise to several alternative splicing variants, among which FGFR2b (mainly expressed in epithelial cells) and FGFR2c (in mesenchymal cells) are the most abundant (reviewed in [159]). FGFR2b and FGFR2c differentially bind specific sets of FGF ligands [160, 161]. Germline *Fgfr2b*-knockout mice die shortly after birth due to multiple-organ abnormalities [162, 163]. Gain of function for *FGFR2* via amplification, mutation, or other mechanisms is found in multiple tumors including gastric [164], lung [165], breast [166], ovarian [167] [168] cancers, and in cholangiocarcinoma [169].

Very little is known regarding the function of FGFR2 in neuroblastoma. The *FGFR2* gene is localized to the 10q26.13 locus. A high frequency of 10q loss of heterozygosity (LOH)—including *FGFR2*—has been described in different tumors ([170–172]). Importantly, 10q LOH has also been described in familial neuroblastomas [173]. In this context, Lázcoz et al. [174] analyzed LOH and FGFR2 promoter hypermethylation at 10q in a panel of neuroblastoma tumor samples and cell lines. Although 10q LOH was observed in 18% of the cases, hypermethylation at the FGFR2 promoter was not observed for any tumor sample, while FGFR2 expression was positive in all 12 cell lines included in the study, pointing to the conclusion that FGFR2 downregulation does not seem to be particularly associated with neuroblastoma pathogenesis. More recently, Salm et al. identified FGFR2 as the top candidate in a human kinome-wide RNAi screen to characterize kinases that, when downregulated, sensitize neuroblastoma cells to cisplatin [175]. This work also demonstrated that MYCN activates *FGFR2* transcription. We identified statistically significant correlations between increased *FGFR2* and decreased survival probability in 5 out of 10 neuroblastoma patient datasets, although showing the opposite correlation in one additional cohort (Fig. 2 top subset). While the role of FGFR2 in neuroblastoma drug resistance and pathogenesis remains to be clarified, our analysis, combined with its function downstream of MYCN, suggests FGFR2 may be an effective target for highly potent small molecule inhibitors.

### Conclusions and future prospects

Our systematic analysis of expression data for all members of the RTK superfamily across 10 well-annotated clinical cohorts of neuroblastoma patients highlights a set of important new therapeutic targets. Our methods corroborate previous independent experimental data identifying several RTKs with known roles in neuroblastoma tumorigenesis, stemness, and metastasis, but most importantly, we also reveal additional RTKs not previously associated with this cancer. We present a detailed review of the current literature for the top therapeutic candidates in the context of neural crest differentiation and neuroblastoma pathogenesis. In doing so, and for the sake of conciseness, we have not exhaustively focused on mechanistic insights of RTKs signaling, but prioritized articles showing cellular to *in vivo* characterizations, which provide translationally relevant concepts. Numerous recent efforts to elucidate RTKs activation, their protein interactomes, and crosstalk with other receptors and pathways [41, 42, 176–178] are also helping to define essential new dimensions of RTK biology in neuroblastoma and other cancers.

Consistent robust correlations with survival across patient cohorts for the top-ranked candidate RTKs support our hypotheses regarding their role in modulating tumor aggressiveness, metastasis, and drug resistance. We hope that translational efforts will nominate such RTKs as effective targets to be incorporated in future innovative clinical therapeutics. This work is urgently needed to advance the cure rate for children suffering from high-risk neuroblastoma.

## Methods

### Expression-survival correlations in R2

All the significant Bonferroni-adjusted *p* values (*p* value < 0.05) correlating neuroblastoma patient survival probability to RTK expression for every available probe of every member of the RTK superfamily across 10 publicly available datasets (Kaplan–Meier plots produced in R2: Genomics Analysis and Visualization Platform (http://r2.amc.nl)) were annotated, and each RTK received a score. The score of each RTK was established by assigning 10 points for every dataset showing one or more significant adjusted *p* value (*p* value < 0.05). One additional point was assigned for every additional probe with a significant *p* value within the same dataset, and finally, one, two, or three additional points were assigned for those *p* values < 10^−10^, < 10^−20^ or < 10^−30^, respectively. Finally, positive expression-to-survival correlations were assigned positive values (red to pink; higher survival probability), whereas negative correlations received negative scores (dark to light blue; lower survival probability). We arbitrarily established that each RTK showing a significant Bonferroni-adjusted *p* value in at least 5 independent cohorts would be considered as a candidate for further analysis. Therefore, a minimum score of 50 points (either positive or negative) was determined as the threshold of “significance” for further consideration. Tables are presented as a pseudo-heatmaps, to highlight the most significant correlations. For any given RTK, each dataset might contain 0, 1, or more probes, giving rise to 0, 1, or more possible significant correlations (with their corresponding *p* values).

### Cancer Cell Line Encyclopedia analysis

Expression data of selected genes (Expression 21Q2 Public database) was downloaded from the CCLE (phases I and II), included in the Cancer Dependency Map portal (https://depmap.org/portal) [63–65], and further analyzed with GraphPad Prism 8.3.0 software. To represent expression data in boxplots (box and whiskers, Turkey’s method), cell lines were grouped by primary disease (cancer type) and sorted by decreasing mean of gene expression. A horizontal line within each box marks the median value. A “ + ” within each data group marks the mean value. The *DDR2* vs. *RET* co-expression graph (Fig. 4C) was plotted using the original CCLE plotting option with data grouped by cancer type/subtype.

## Supplementary Information

Below is the link to the electronic supplementary material.

Supplementary file1 (PDF 261 KB)

## Data Availability

Not applicable
