# Supplementary material for: Systematic review of the receptor tyrosine kinase superfamily in neuroblastoma pathophysiology
Source: Cancer Metastasis Rev. 2021 Oct 30;41(1):33–52. doi: 10.1007/s10555-021-10001-7 (PMC8924100; doi:10.1007/s10555-021-10001-7)

Supplementary Figure 1 . Associations of RTK gene expression to neuroblastoma patient survival by phylogenetic classification.

negative correlations
5.0E-02 to 9.9E-04
1.0E-05 to 9.9E-09
<1.0E-10

positive correlations
5.0E-02 to 9.9E-04
1.0E-05 to 9.9E-09
1.0E-10 to 9.9E-19
<1.0E-20

Class	Family	RTK	786 Cangelosi	649 Kocac			498 SEQC RPM					498 SEQC custom				283 Primary NRC		251 Oberthuer		249 Asgharzadeh		102 Seeger			101 Maris		88 Versteeg				score
I	EGFR	EGFR										8,5E-04										2,6E-03	2,9E-02	2,5E-03							-2
		ERBB2		7,7E-03			1,7E-04	1,9E-04				5,7E-05	3,3E-03					4,5E-07							2,2E-02						32
		ERBB3*	2,6E-08	2,8E-05	4,9E-08		2,8E-09	6,8E-06				1,3E-07	1,2E-07	1,3E-03		1,5E-02						4,8E-02					2,0E-07				54
		ERBB4																				2,3E-02									-10
II	InsR	IGF1R	3,5E-06	8,6E-08	9,3E-03	2,5E-03	5,3E-04					6,8E-09	2,0E-06	3,0E-03	1,8E-05			9,2E-03													55
		INSR										5,7E-04						6,4E-12													21
		INSRR		3,1E-06			4,7E-19					1,2E-14				2,5E-16		4,0E-02		6,1E-03							3,5E-05				73
III	PDGFR	PDGFRA	4,2E-06	4,6E-03	1,6E-03	8,0E-05	7,6E-03					3,9E-04	1,8E-03			6,0E-07						2,6E-02			2,4E-02		8,0E-05	1,1E-04	2,5E-03		-85
		PDGFRB					1,6E-02											2,1E-13	2,3E-02			3,9E-03									12
		CSF1R	2,5E-07	1,8E-06			8,6E-06					9,8E-07						1,3E-04				2,8E-02									40
		KIT	2,2E-07	2,1E-03			6,2E-04	8,4E-04				8,0E-05				1,3E-02		8,8E-03									1,3E-02				-71
		FLT3	4,5E-02	2,0E-07			3,1E-04					1,7E-06																			40
IV	VEGFR	FLT1					8,9E-06	1,3E-03	2,0E-02	3,5E-02		7,9E-05	2,1E-05												4,5E-02		8,0E-04	2,4E-02			8
		KDR	6,7E-03	1,1E-02			1,6E-03					3,3E-03	2,3E-03					1,7E-07													51
		FLT4										2,6E-02	4,8E-03														5,7E-03	1,4E-02	6,3E-03		-1
V	FGFR	FGFR1																				1,0E-04	4,7E-04	1,0E-03			3,8E-05	9,5E-06	2,5E-04	1,1E-02	-25
		FGFR2	6,6E-04	1,4E-03	6,2E-08		6,8E-03	2,0E-02	1,2E-02	2,3E-02	1,1E-02					1,0E-02						8,6E-03	3,6E-02		9,5E-03						-50
							1,5E-02	2,6E-02	5,2E-04	2,8E-04																					
		FGFR3		3,6E-02												2,2E-06		4,9E-04													-30
		FGFR4					1,1E-03	2,9E-03	1,2E-03			2,7E-03																			22
VI	CCK	PTK7*	1,7E-05													1,0E-02									2,7E-02		2,4E-02				-20
VII	TRK	NTRK1	3,4E-33	6,0E-23	4,5E-22		5,7E-23	4,2E-23	1,2E-22			2,4E-23	2,3E-23			2,4E-17	2,5E-16	2,9E-10	9,5E-12	5,5E-03	6,1E-03	4,5E-05			3,5E-05	1,6E-05	2,7E-10				130
		NTRK2										1,0E-02	9,6E-03			3,5E-02		3,2E-24				4,2E-04	1,3E-03	3,3E-03	8,8E-04		6,0E-03	1,2E-02			-21
		NTRK3		1,1E-03								9,9E-03	2,2E-02					1,4E-03				1,9E-02	3,1E-02								42
VIII	HGFR	MET	5,6E-11	8,7E-08			5,8E-09	2,7E-09				4,7E-07				3,4E-02		2,4E-02				8,5E-04	1,9E-03	5,2E-04							-54
		MST1R	3,0E-16	6,4E-12			1,2E-18	6,1E-19				6,0E-16						9,3E-12													56
IX	EphR	EPHA1		1,2E-03			1,3E-06					2,1E-04																			30
		EPHA2	4,7E-02				2,4E-04					1,6E-03																			30
		EPHA3																				1,8E-02									-10
		EPHA4	2,5E-02	2,6E-03	1,9E-02		1,3E-02					8,8E-04	2,0E-06	6,2E-05																	43
		EPHA5	1,1E-20	8,2E-13	4,0E-12		1,8E-14	1,7E-14				3,3E-16	6,5E-17			5,5E-12		6,0E-08	3,7E-07	3,4E-02					2,5E-02		1,1E-06	4,2E-08			104
		EPHA6		1,2E-04								2,0E-06								4,3E-03											30
		EPHA7	2,5E-02				1,1E-02					9,0E-04	1,9E-03			6,5E-03									2,1E-03		1,1E-02	6,6E-05			62
		EPHA8																4,2E-05													10
		EPHA10*	3,7E-12	3,6E-15			7,8E-11					6,3E-15				6,1E-04											3,2E-02	1,9E-02			65
		EPHB1														8,8E-04						1,6E-04					7,1E-03	7,8E-05	2,5E-02		-32
		EPHB2																5,8E-03							3,8E-02		2,1E-02				10
		EPHB3	4,2E-03	5,8E-04			5,4E-06					4,3E-05						1,9E-03									2,6E-02				60
		EPHB4		3,3E-03														4,1E-04				6,9E-03									-30
		EPHB6*	2,0E-08	1,0E-03			4,6E-06					5,4E-04						1,4E-02							2,3E-03						60
X	AXL	AXL	1,4E-06	5,5E-10			2,5E-04	3,9E-04				1,0E-02	1,4E-10					2,6E-05				1,0E-03									44
		MERTK	4,2E-04	1,1E-04								1,3E-04	3,4E-02			4,7E-04															41
		TYRO3	2,6E-03				3,9E-03	1,3E-03				4,0E-02				1,3E-02															-20
XI	TIE	TIE1														2,8E-02									7,8E-03						-20
		TEK	1,9E-05	5,9E-03			3,7E-04					4,3E-05				2,9E-03															30
XII	RYK	RYK*	7,6E-11	3,0E-05			2,1E-11	4,2E-11				4,8E-03										6,0E-03	2,4E-02		2,6E-02						-65
XIII	DDR	DDR1		2,9E-02	3,2E-02		2,0E-02	1,7E-02	9,4E-03																						26
							7,1E-03	9,4E-03	1,2E-02																						
		DDR2	1,2E-15	1,4E-13	2,0E-10		3,0E-12	1,0E-11				7,7E-08	7,6E-11			4,0E-09		1,1E-06		7,4E-03		1,1E-04					4,1E-05	1,1E-07	3,7E-05	1,4E-02	-102
XIV	RET	RET	5,4E-14	1,0E-11	6,4E-16		3,2E-11	1,7E-11				1,7E-08	1,3E-12			4,3E-05		8,8E-07	2,9E-05			2,3E-02			2,9E-06		1,1E-02	4,2E-08	3,5E-03		-102
XV	ROS	ROS1	9,2E-09				1,4E-08					1,0E-02																			10
XVI	LTKR	LTK					1,3E-04	5,0E-04	3,2E-04			2,8E-10																			23
		ALK	2,7E-08	2,4E-06			4,0E-04					3,9E-04				1,1E-03		8,9E-04							2,3E-02						-70
XVII	ROR	ROR1*					4,6E-02					2,9E-02				3,8E-02															-10
		ROR2*	4,2E-09	7,1E-05			8,2E-03									2,1E-04						7,3E-03					3,9E-02				-60
XVIII	Musk	MUSK		7,6E-03			1,9E-03	1,6E-03	5,2E-04											1,2E-02											12
XIX	LMR	AATK	8,1E-05	6,7E-03			5,6E-05	1,2E-04				1,8E-05	1,2E-03	1,3E-02																	43
		LMTK2		7,9E-05																											-10
		LMTK3	1,4E-10	9,1E-12			3,2E-12					4,0E-08																			43
XX	STYK	STYK1*	2,5E-02	1,8E-03																		1,4E-02									-30

Supplementary Figure 2.

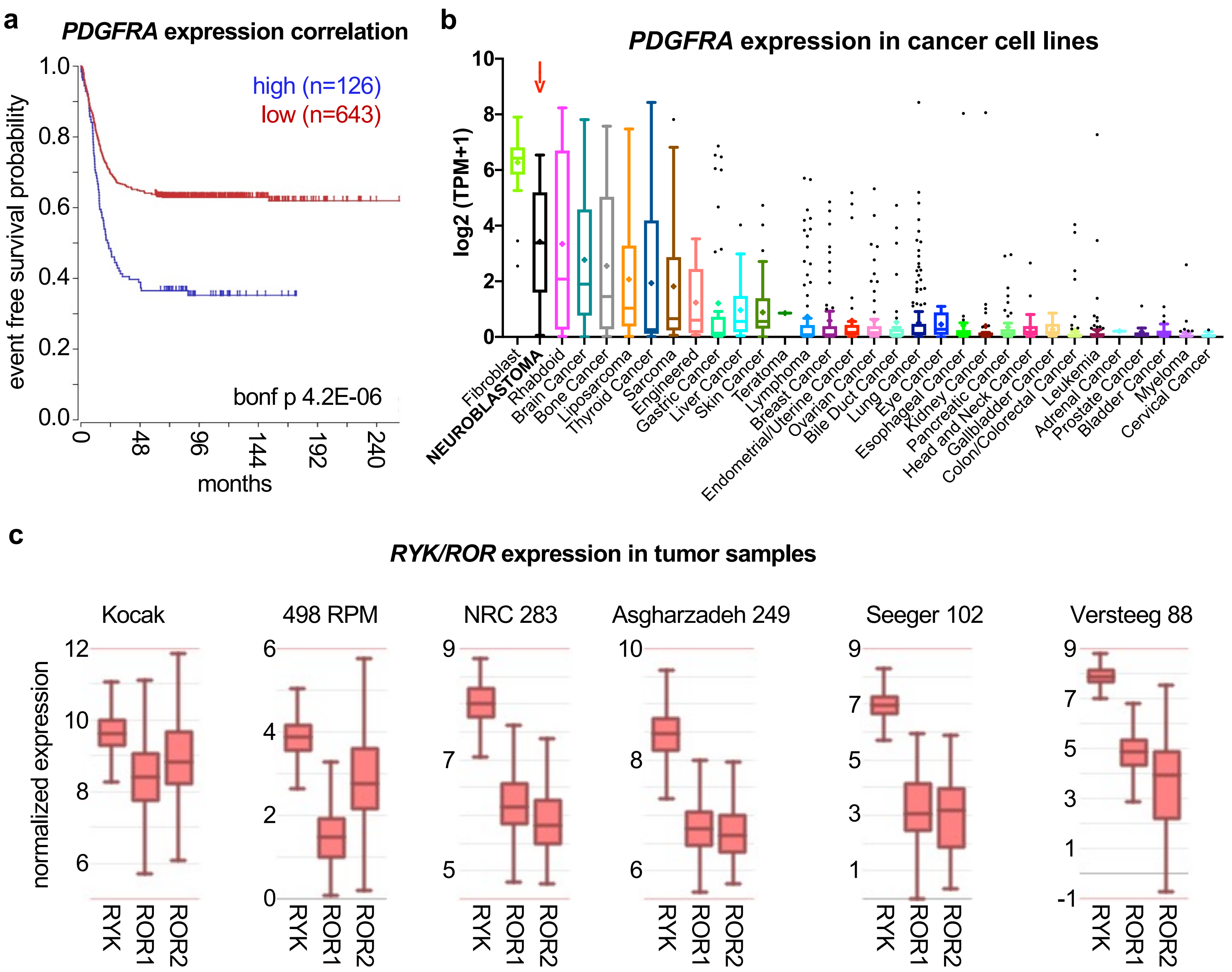

Supplement: Supplementary file 1 — Supplementary file1 (PDF 261 KB) [file 10555_2021_10001_MOESM1_ESM.pdf]
